# Neonatal Multisensory Processing in Preterm and Term Infants Predicts Sensory Reactivity and Internalizing Tendencies in Early Childhood

**DOI:** 10.1007/s10548-020-00791-4

**Published:** 2020-08-12

**Authors:** Nathalie L. Maitre, Alexandra P. Key, James C. Slaughter, Paul J. Yoder, Mary Lauren Neel, Céline Richard, Mark T. Wallace, Micah M. Murray

**Affiliations:** 1 Center for Perinatal Research at the Abigail Wexner Research Institute, Nationwide Children’s Hospital, Columbus, OH USA; 2 Department of Hearing and Speech Sciences, Vanderbilt University Medical Center, Nashville, TN USA; 3 Vanderbilt Kennedy Center, Vanderbilt University Medical Center, Nashville, TN USA; 4 Department of Biostatistics, Vanderbilt University Medical Center, Nashville, TN USA; 5 Department of Special Education, Peabody College of Education and Human Development, Vanderbilt University, Nashville, TN USA; 6 Departments of Psychology and Pharmacology, Vanderbilt University, Nashville, TN USA; 7 Department of Psychiatry and Behavioral Sciences, Vanderbilt University Medical Center, Nashville, TN USA; 8 Vanderbilt Brain Institute, Vanderbilt University, Nashville, TN USA; 9 The Laboratory for Investigative Neurophysiology (The LINE), Department of Radiology, University Hospital Center and University of Lausanne, Lausanne, Switzerland; 10 Sensory, Perceptual, and Cognitive Neuroscience Section, Center for Biomedical Imaging (CIBM) of Lausanne, Lausanne, Switzerland; 11 Department of Ophthalmology, Fondation Asile des aveugles and University of Lausanne, Lausanne, Switzerland; 12 Department of Pediatrics, Nationwide Children’s Hospital, 700 Children’s Way, Columbus, OH 43205 USA

**Keywords:** Premature, Infant, Neonate brain, EEG, Multisensory, Touch, Sound, Hypersensitivity

## Abstract

Multisensory processes include the capacity to combine information from the different senses, often improving stimulus representations and behavior. The extent to which multisensory processes are an innate capacity or instead require experience with environmental stimuli remains debated. We addressed this knowledge gap by studying multisensory processes in prematurely born and full-term infants. We recorded 128-channel event-related potentials (ERPs) from a cohort of 55 full-term and 61 preterm neonates (at an equivalent gestational age) in response to auditory, somatosensory, and combined auditory-somatosensory multisensory stimuli. Data were analyzed within an electrical neuroimaging framework, involving unsupervised topographic clustering of the ERP data. Multisensory processing in full-term infants was characterized by a simple linear summation of responses to auditory and somatosensory stimuli alone, which furthermore shared common ERP topographic features. We refer to the ERP topography observed in full-term infants as “typical infantile processing” (TIP). In stark contrast, preterm infants exhibited non-linear responses and topographies less-often characterized by TIP; there were distinct patterns of ERP topographies to multisensory and summed unisensory conditions. We further observed that the better TIP characterized an infant’s ERPs, independently of prematurity, the more typical was the score on the *Infant/Toddler Sensory Profile* (ITSP) at 12 months of age and the less likely was the child to the show internalizing tendencies at 24 months of age. Collectively, these results highlight striking differences in the brain’s responses to multisensory stimuli in children born prematurely; differences that relate to later sensory and internalizing functions.

**Electronic supplementary material:**

The online version of this article (10.1007/s10548-020-00791-4) contains supplementary material, which is available to authorized users.

## Introduction

Despite decades of results on how information from the different senses is combined, we still know surprisingly little about how such multisensory processes develop in humans (Bremner et al. 2012; Murray et al. 2016). Nonetheless, it is recognized that the construction of a unified perceptual reality through multisensory interactions is essential for the establishment of concepts such as self vs. non-self, critical to social-emotional functioning (Aspell et al. 2012; Bahrick and Lickliter 2012; Lewkowicz 2014; Lickliter and Bahrick 2004; Rochat et al. 2012). During the neonatal period, when peripheral and central nervous systems are still developing, integration of lower-level physical stimulus features (such as duration, rhythm and intensity) is a first critical step in the maturation of multisensory processes (Murray et al. 2016). Theoretically, without the ability to integrate multiple streams of lower-level sensory attributes, an infant cannot interpret these stimuli and is left with perceiving the environment as confusing, distracting or even potentially hostile (Lewkowicz 2014; Lickliter 2011).

There continues to be ongoing discussion and debate as to whether the development of multisensory processes is innate (though undoubtedly also tuned by environmental experiences) or instead arises after the child has accrued at least several months of experiences with the sensory world (Dionne-Dostie et al. 2015). While objective and quantitative studies in neonates and infants are challenging, a seminal study by (Lewkowicz and Turkewitz 1980) measured 3–4 week-old infants’ heart rates and showed that these children can associate light and sound intensities. More recent results have shown that newborn infants can match numerosity across the senses (Izard et al. 2009) and that 4-month-old infants are sensitive to the spatial congruence of auditory-tactile events (Thomas et al. 2018). However, one constraint of most studies in infants and young children is that they are typically based on child-appropriate behavioural measures, such as preferential looking, and consequently provide limited insights into the putative neurobiological mechanisms and maturation of multisensory processes. One pilot study, using MEG in a set of 9 infants as young as 6-months-old, demonstrated there to be non-linear auditory-somatosensory neural response interactions, defined as the difference in responses to simultaneous presentations of multisensory pairs versus the summed responses to auditory and somatosensory stimuli alone. Such responses were found to be more pronounced in 11–13 than in 6–9 month-olds (Stephen et al. 2007).

Thus, in light of the current knowledge gap concerning neonatal multisensory processes, the first objective of the present study was to record high-density EEG in neonates during auditory, tactile, and multisensory (i.e., audio-tactile) stimulus presentation and thus characterize the presence and core characteristics of multisensory neural response interactions.

The second objective of this study was to determine the role played by early-life experiences in shaping multisensory processes, contrasting cases of full-term and premature births. The nature of the sensory experiences received during the perinatal period can have a wide-ranging impact on later development across domains from sensory processing to higher cognitive function (Maitre et al. 2017; Nelson 2001; Sheridan and Nelson 2009; Thompson and Nelson 2000). In addition, the impact of sensory experiences undoubtedly extends into the gestational period, which likely has significant impact in cases when infants are born prematurely and spend all or some period of the final trimester in a neonatal intensive care unit (NICU) (Eckstein Grunau 2002; Foster and Verny 2007; Lickliter 2011). Of concern, therefore, is the fact that preterm infants are exposed for weeks or months to atypical sensory stimuli in the NICU (Santos et al. 2015). Preterm infants are exposed to few typical stimuli such as human voice, and there is some evidence that this may result in maturation of voice perception (Adam-Darque et al. 2020) at term equivalent age, as compared to infants born full-term. However, parental involvement in NICUs varies widely, and the majority of stimuli in NICUs neither approximate intrauterine nor home, infant-directed environments (Lahav and Skoe 2014; Oller et al. 2019; Best et al. 2018). After months cared for in the NICU, preterm speech sound discrimination and somatosensory processing appear to be worse than in term-born counterparts at discharge to home. Past research has also demonstrated that the effects of immaturity on sensory system development are not fully compensated in the first years (Brummelte et al. 2012; Key et al. 2012; Rose et al. 2008). Evidence for this comes from the diminished brain responses of preterm infants in response to sensory stimuli at discharge, with long-lasting developmental consequences (Key et al. 2012), including predicting worse developmental outcomes in toddlerhood (Maitre et al. 2013).

The third objective of this study was to then use an EEG-based measure of multisensory processes in neonates as a predictor of later outcome measures when these children were infants and toddlers (i.e., at 12 and 24 months, respectively). The consequences of preterm birth combined with atypical sensory experiences may result in behaviorally-measured sensory processing problems, which are themselves later associated with concerns for behavioral adaptation and social emotional functioning (Cassiano et al. 2016). Infants with atypical sensory neurological thresholds to the home environment are challenged in their social and emotional interactions: they can require abnormally high levels of concurrent sensory stimuli to experience an interaction or they can become overwhelmed at low levels of stimulation diversity and intensity. These maladaptive responses are associated with worse behavioral outcomes in preterm and term infants (Machado et al. 2017). Early atypical neurobehaviors associated with behavioral sensory hypo- and hyper-responsiveness in later childhood can be detected at discharge from the hospital (Ryckman et al. 2017). Behavioral risk tendencies in early childhood are often precursors of behavioral disorders later in adolescence or adulthood, such as anxious, withdrawn, or fearful (internalizing) behaviors and aggressive, antisocial (externalizing) behaviors (Achenbach 2009). Preterm infants are at higher risk for internalizing and externalizing behaviors (Bhutta et al. 2002), and ensuing problems at adolescence and adulthood (Hack 2009; Hille et al. 2008; Turpin et al. 2019). This objective is also predicated on a growing literature in schoolchildren (Barutchu et al. 2011, 2019a, b, c; Birch and Belmont 1965; Denervaud et al. 2020; Rose et al. 2008, 2012) as well as older infants and toddlers showing that multisensory processes, in particular, are predictive of the integrity of various facets of cognitive functions, including memory, attention, executive functions, and fluid intelligence.

In particular, the construction of auditory-tactile processing in infants and its later associations with atypical sensory thresholds and maladaptive behaviors such as excessive internalizing and external tendencies. Therefore, a knowledge gap in the development of multisensory processes exists, with little neurophysiological evidence regarding the construction of multisensory representations during the neonatal period, or how these representations are altered by early birth and the sensory environment experienced by the premature baby. Additionally, testing whether there are differences in the behavioral outcomes of infants with varying multisensory responses may suggest possible associations between later adaptations to sensory environments and behavioral tendencies similar to those studied in older children. Ultimately, understanding the construction of multisensory responses in infancy is necessary to rational design of optimal sensory environments and interventions addressing poor social-emotional and behavioral outcomes in childhood.

## Methods

### Participants

A cohort of healthy preterm and full-term infants was recruited from the newborn nursery and the Neonatal Intensive Care Unit at Vanderbilt University Medical Center (Nashville, TN, USA) (Table 1). These infants are the same as those previously reported (Maitre et al. 2017). The Institutional Review Board approved the study. Inclusion criteria were preterm infants < 37 weeks gestational age (GA) at birth and full-term infants ≥ 37 weeks GA. Exclusion criteria were lethal congenital abnormalities or severe abnormalities on any cranial imaging (cerebellar hemorrhage, intraventricular hemorrhage grade III or IV, periventricular leukomalacia, ischemia or stroke) or infants who had failed their auditory brainstem response testing performed at 34 weeks postmenstrual age (PMA).

**Table 1:** Participants’ characteristics and outcomes

Demographics			
	All	Full-term (N = 55)	Preterm (N = 61)
Gestational age, weeks, median (IQR)	36 (31, 39)	40 (39, 41)	31 (30, 33)
Sex (% female)	52	49	54
PND at testing, median (IQR)	5 (2, 28)	2 (1, 2)	28 (11, 40)
Maternal education (% high school graduation)	95	95	94

One-year ITSP outcomes			
	All	Full-term (N = 42)	Preterm (N = 49)
Corrected age at testing, months, median (IQR)	13 (12, 14)	13 (12, 14)	13 (12, 15)
Atypical (%)	20	21	18

Two-year CBCL outcomes			
		Full-term (N = 34)	Preterm (N = 44)
Corrected age at testing, months, median (IQR)	24 (24, 26)	25 (24, 26)	24 (23, 25)
Internalizing: above risk threshold of 16 (%)	65	55	72
Externalizing: above risk threshold of 20 (%)	11	6	16

*IQR* Interquartile range, *PND* postnatal days, *ITSP* Infant/Toddler Sensory Profile, *CBCL* Child Behavior Checklist

### General Procedures and Variables

#### Overview

Continuous EEG data were acquired using published protocols as near to discharge as possible in preterm infants (35–38 weeks PMA) and 1–3 days after birth for full-term infants. Primary caregivers completed the *Infant/Toddler Sensory Profile* (ITSP) (Dunn 2002) at 12–15 months corrected age (CA). The *Child Behavior Checklist* (CBCL) was completed at 24–27 months CA (Achenbach 2009; Dunn 2002). Primary caregivers received assistance from a trained therapist, as needed, to complete the assessments. In accordance with IRB regulations, all data were stored in a secure Research Electronic Data Capture (REDCap) encrypted server (Harris et al. 2019).

#### EEG Acquisition and Pre-processing

**Fig. 1:**
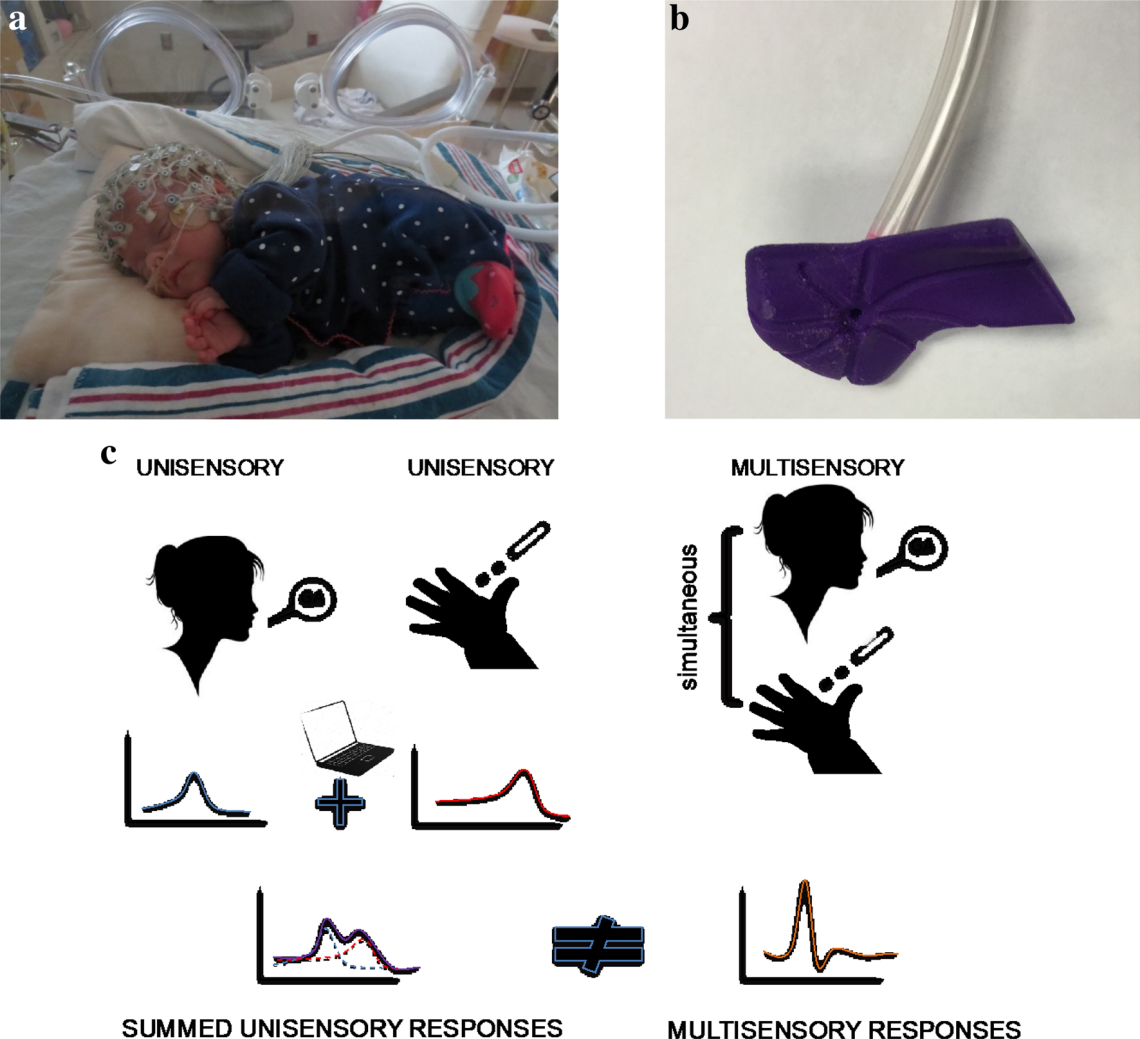
Neonates were tested using a 128-electrode EEG net in their isolettes **a** if they were in the NICU (preterm) or in their bassinettes in the newborn nursery (full-term). Calibrated tactile stimulation was delivered via a custom 3D-printed holder fastened with a neoprene glove to the infant’s hand allowing the air pulse to always have the same pressure and target (**b**). The paradigm presents stimuli in random order and at random intertrial intervals, with a standard /ga/ sound and the tactile air puff stimulation presented separately for unisensory conditions or simultaneously for multisensory conditions (see **c**). Finally, in the analysis, the unisensory responses are algebraically added to produce a summed response and compared with the true multisensory response (panel **c**, lower)

A high-density array net of 128 electrodes embedded in soft sponges (Geodesic Sensor Net, EGI, Inc., Eugene, OR, USA) continuously recorded the EEG using Net Station software (v. 4.3; EGI, Inc.) (Fig. 1). Data were sampled at 1000 Hz. All infants were tested in his/her patient room while lying on their backs in the bassinet/crib or being held in the supine position by a caregiver. No restraint was used, and infants were tested in quiet alert states. ERP responses were collected in response to 50 randomly presented trials for each of four stimulus conditions. Intertrial intervals randomly varied between 2500 and 3000 ms between any trial, with no more than two events including a tactile stimulus presented in a row, to prevent tactile habituation (Maitre and Key 2014).

There were four stimulus conditions. The “tactile” condition was an air puff emanating from a nozzle that was positioned 5 mm above the skin of the palmar surface of the right hand using a mold holder. The puff delivered a calibrated pressure of 5 psi (34.5 kPa) over a 3 mm^2^ area, which approximates light touch. The device emitting the air puff also produced a mechanical sound as air exits the system through an alternate matching nozzle positioned away from the subject. The “sham puff” condition entailed only the mechanical sound of the device producing the air puff. That is, a second nozzle was pointed away from the infant’s hand. This condition provided a control for the auditory stimulus that occurred when the air puff was delivered to the hand. The “auditory” condition involved presentation of a computer-synthesized /ga/ syllable from the “transition only” stimulus series employed by (Stevens and Blumstein 1978). The stimulus had no initial noise burst. The speech sound was synthesized on a Klatt (cascade) synthesizer so that the amplitude of the formant was modulated as a function of the respective formant frequencies, as in natural speech. Sound was delivered at 60 dBA using a speaker 12 cm at midline above the infant’s head. The “sham puff” condition was delivered simultaneously and synchronously as the /ga/, allowing for the auditory condition to be compared with tactile and multisensory conditions. The “multisensory” condition was the simultaneous and synchronous delivery of the “tactile” and “auditory” conditions. Time-locking of simultaneous stimuli under 5 ms was confirmed using oscilloscopes as previously described (Maitre and Key 2014).

Data pre-processing and ERP analyses were implemented with the Cartool freeware (Brunet et al. 2011). Prior to epoching, the continuous EEG was filtered (low-pass 40 Hz; high-pass 0.3 Hz; using a second-order Butterworth filter with 12 dB/octave roll-off that was computed linearly in both forward and backward directions to eliminate phase shifts). Peri-stimulus epochs of continuous EEG spanned from 100 ms pre-stimulus onset to 500 ms post-stimulus onset. Artifact-contaminated epochs were identified via automated routines in Net Station and confirmed with visual inspection for movement, eye blinks and eye movements, as well as other sources of transient noise. An infant was deemed to have analyzable ERP data whenever there were more than 10 usable trials per each condition, with every usable trial also having more than 108 of 128 electrodes with artifact-free signals. Mean numbers of usable trials per conditions were however greater than the pre-specified minimum (multisensory condition 22.3 ± 8.6; tactile conditions 23.2 ± 8.8; auditory 22.8 ± 8.9). ERPs were the result of signal averaging epochs for each condition. Data at artifact-contaminated electrodes in the ERP were interpolated using 3D splines (Perrin et al. 1987). ERP data were then baseline-corrected using the 100 ms pre-stimulus interval and re-referenced to the common average reference.

##### ITSP

The ITSP consists of a 48-item questionnaire assessing how and how often the child responds to different sensory experiences in the home environment (Dunn 2002). One key variable derived from this instrument is the infant’s sensory reactivity, which is defined as atypical if it was either lower or higher than a standard range. ‘Lower than typical’ reflects an infant for whom minimal sensory input causes the infant to respond and ‘higher than typical’ reflects an infant for whom large amounts of stimulation are required before the infant responds (Dunn 2002). Typical and atypical ranges are provided for scores in the 7–12-month age band using published norms, generating a dichotomous variable. Using these norms, we classified infants into typical or atypical sensory reactivity subgroups.

##### CBCL

Scores were obtained for internalizing and externalizing tendencies on the CBCL and risk group member was assigned using the Achenbach System of Empirically Based Assessment (ASEBA) manual (Achenbach and Rescorla 2000). Internalizing tendencies include anxiety, depression, withdrawn, and somatic complains. Using the guidelines accompanying the instrument, a cut-off of 16 on the internalizing items was used to classify infants into an “internalizer” subgroup or not. Externalizing tendencies include rule-breaking and aggressive behaviors. A cut-off of 20 on the externalizing items was used to classify infants into an “externalizer” subgroup or not. Internalizing and externalizing problems in early childhood are associated with specific psychopathology in adolescence and adulthood (Achenbach 2009; Hofstra et al. 2018, 2002).

## Results

Of 116 infants tested in the NICU and newborn nursery, 78.5% were followed to 12 months CA and 68.1% were followed to 24 months CA. Full-term and preterm infants had similar rates of atypical sensory threshold in the home (Table 1).

### ERP Results

**Fig. 2:**
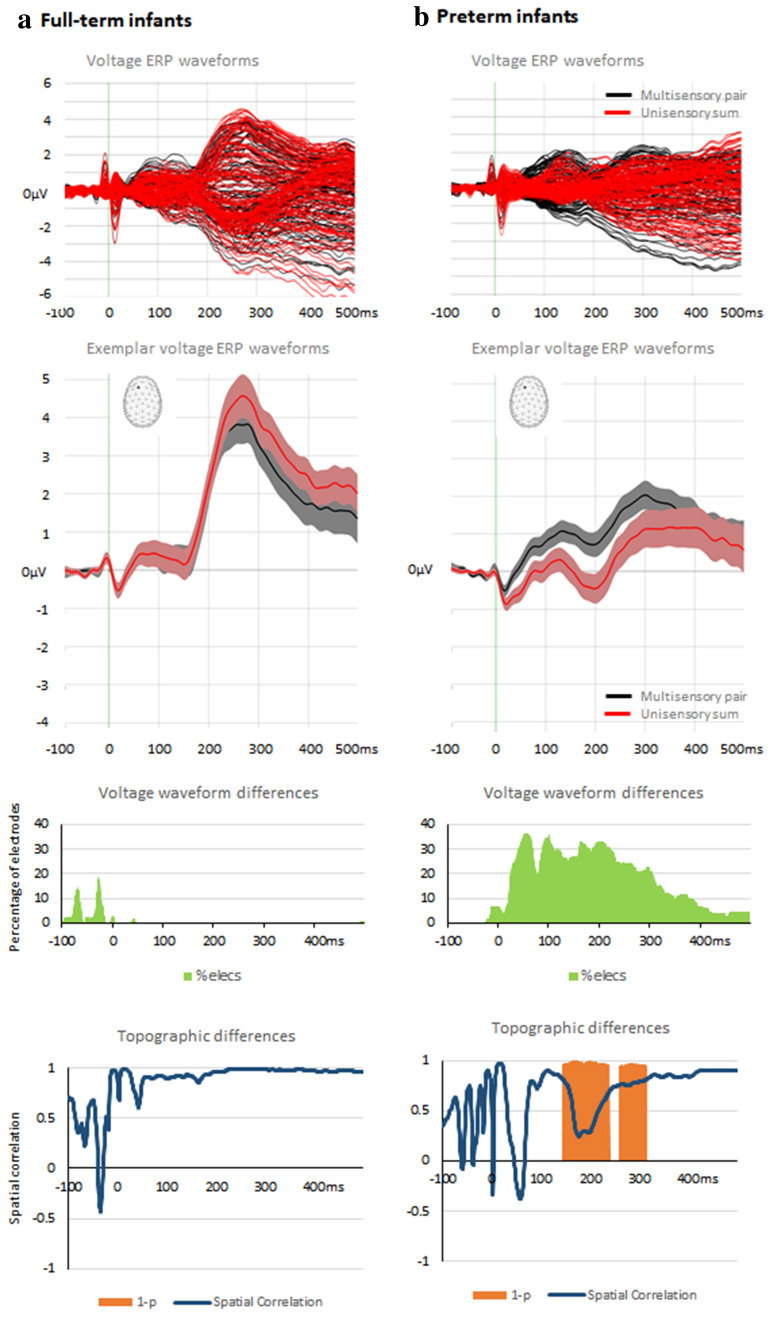
ERP analyses contrasting multisensory and summed unisensory responses from full term and preterm infants (panels **a** and **b**, respectively). The uppermost row displays group-averaged data from all electrodes for the multisensory pair and summed unisensory conditions. The next row displays group-averaged data (s.e.m. indicated by shaded area) from an exemplar fronto-central electrode (see inset). The next row displays the percentage of the electrode montage exhibiting a significant difference as a function of time. The bottom row displays the spatial correlation between multisensory and summed unisensory responses (blue line) as well as time periods of significant differences (orange areas)

Our first research aim was to ascertain whether infants exhibit non-linear neural response interactions to auditory-tactile multisensory stimuli and, if so, to determine if this followed from modulations in response strength and/or response topography; the latter of which would be indicative of changes in the configuration of the underlying brain sources (reviewed in Biasiucci et al. 2019; Tivadar and Murray 2018). To achieve this, we compared ERPs to multisensory stimulus pairs with summed ERPs to the corresponding unisensory conditions (see Foxe et al. 2000; Murray et al. 2005; Sperdin 2009) for similar analyses of auditory-somatosensory interactions in adults). Specifically, we ran two sets of paired *t*-tests—i.e. one per group—comparing multisensory pair and summed unisensory ERPs at each scalp site as a function of time (Fig. 2). We then compared global field power, a measure of response strength, in a similar way and using a paired *t*-test as a function of time. Finally, we tested for topographic differences using global dissimilarity and a non-parametric permutation test. In all analyses, effects were considered reliable if significant differences were observed for a minimum of 20 ms contiguously (Guthrie and Buchwald 1991; Murray et al. 2018). For full-term infants there was no evidence of neural response differences between paired multisensory and summed unisensory conditions in either global field power or global dissimilarity. By contrast, for preterm infants, there were robust topographic differences between multisensory pair and summed unisensory conditions during the 143–240 ms post-stimulus time period as well as 258–315 ms post-stimulus time period. There was no evidence of sustained modulations in global field power between paired vs. summed conditions in the preterm infants. We would also note that there were also differences at the level of voltage ERP waveforms beginning at approximately 40 ms post-stimulus onset, which is generally consistent in their timing with effects in adults (Murray 2005) and children (Brett-Green et al. 2008). However, we would remind readers that analyses of voltage ERP waveforms are reference-dependent and that the presence, timing and spatial distribution of significant differences will vary with the reference choice (cf. Murray et al. 2008; Tzovara et al. 2012; Tivadar and Murray 2018 for discussion). We therefore focus our interpetations on reference-independent measures; i.e. global field power and dissimilarity. This collective pattern would indicate that for preterm infants there are distinct brain networks responding to multisensory versus unisensory stimuli, whereas there was no evidence for such differences in full-term infants.

Our next research aim was to ascertain if and when temporally stable networks of brain activity engaged by infants differ as a function of preterm status and/or stimulus condition. To address this research aim, we used a multistep process. First, we applied an unsupervised hierarchical cluster analysis to the group-averaged (regardless of preterm status) ERP data that was concatenated across responses to the multisensory and summed unisensory tactile and auditory conditions. This clustering identified the minimal number of microstates, which are represented as temporally stable patterns of brain activity at the scalp (hereafter template maps), to account for the maximal global explained variance in the entire ERP dataset. In the present case, 16 clusters that involved 12 different template maps yielded a global explained variance of 96.3% (see Supplementary Fig. 1).

**Fig. 3:**
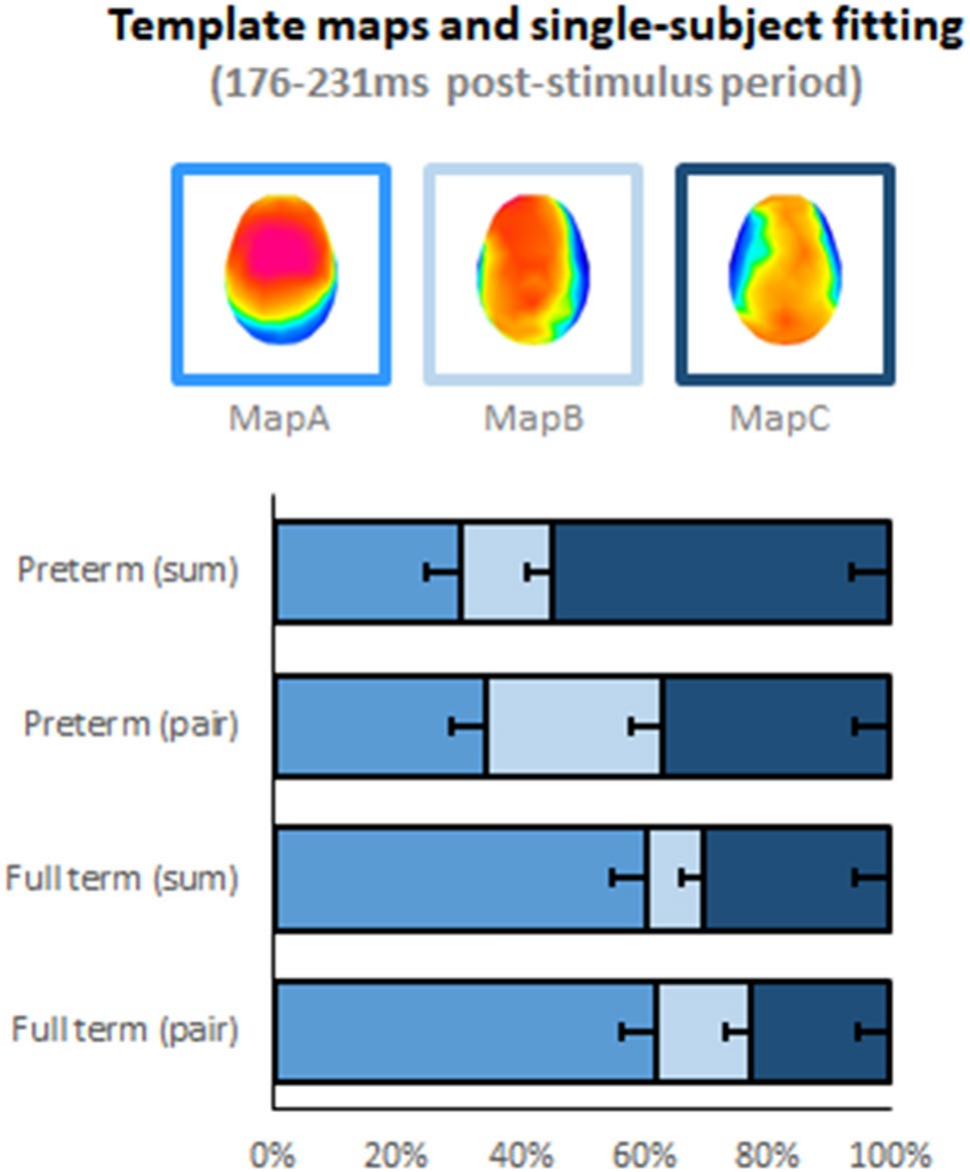
Template maps identified over the 176–231 ms post-stimulus period via unsupervised hierarchical clustering of the ERP topography using the group-averaged dataset concatenated across conditions and full-term and preterm infants. The maps are displayed with the nose upwards and left hemiscalp on the left. The bar graphs display the single-subject fitting based on the spatial correlation between the template maps shown the top row and each time point of the infant’s data over the 176–231 ms post-stimulus period. The bars show the average percentage of time each template map yielded the highest spatial correlation (error bars indicate s.e.m.)

Second, after re-ascribing preterm status as well as multisensory and summed unisensory condition labels back onto the template maps, it is possible to generate data-driven hypotheses regarding time windows when different template maps appear to better characterize either preterm status and/or condition. There were two time windows during which the microstates characterizing the group-averaged data appeared different: 176–231 and 232–375 ms. During each of these time windows, three different template maps primarily characterized the brain responses in the group-averaged data. These were labeled Maps A–C for the earlier time window and Maps D-F for the later time window (Fig. 3 and Supplementary Fig. 2, respectively).

Third, for each time window separately, we calculated the spatial correlation between each template map and each infant’s ERP data from the multisensory and summed unisensory conditions. These spatial correlations indicate for each time frame which of the three template maps best matches the brain response from each participant and each condition. From these within-participant spatial correlations, we computed the proportion of a temporal window characterized by each template map by each condition.

Fourth, these proportions were then submitted to a mixed-model ANOVA using Preterm Status (preterm vs. full-term) as the between-subjects factor and both Condition (multisensory vs. paired unisensory) and Map (either Maps A-C or Maps D-F, depending on the time window analyzed) as the within-subjects factors. This analysis allowed us to determine if there were topographic differences between conditions (by extension differences in active brain networks) that varied as a function of preterm status. It also allowed us to quantify the degree to which topographic patterns in the preterm infants resembled those observed in full-term infants. Presumably, the pattern and preponderance of template maps during stimulus processing in full-term infants is more adaptive than those observed in preterm infants [see also (Maitre et al. 2017) for the case of brain responses to light touch].

For the earlier time window (176–231 ms), Maps A–C were fitted to the data (Fig. 3). The ANOVA revealed a significant main effect of Map (F_(1.67,190.45)_ = 17.96; *p* < 0.001; η_*p*_^2^ = 0.14), a significant Condition × Map interaction (F_(1.86,211.71)_ = 4.59; *p* = 0.013; η_*p*_^2^ = 0.04), as well as a significant Preterm Status × Map interaction (F_(1.67,190.45)_ = 12.72; *p* < 0.001; η_*p*_^2^ = 0.10). The 3-way interaction was not significant (F < 1). In light of the Condition × Map interaction, indicating that each condition was better characterized by maps to varying degrees as well as the absence of a significant 3-way interaction, we next performed 1-way ANOVAs for each condition separately and collapsing across Preterm Status. For the multisensory pair condition, there was an effect of Map (F_(1.87,215.54)_ = 7.94; *p* < 0.001; η_*p*_^2^ = 0.07). Map A predominantly characterized responses to multisensory stimulus pairs. On average, Map A best correlated with individual infant’s brain responses 47.7 ± 4.2% of the time (mean ± s.e.m.), which was significantly more than either Map B (22.3 ± 3.3%; t_(115)_ = 3.91; *p* < 0.001) or Map C (30.0 ± 3.8%; t_(115)_ = 2.43; p < 0.02); the latter two of which did not differ (p > 0.18). For the summed unisensory condition, there was an effect of Map (F_(1.58,181.24)_ = 15.58; *p* < 0.001; η_*p*_^2^ = 0.12). Map A and Map C characterized responses 44.8 ± 4.3% and 43.1 ± 4.3% of the time, respectively, with no difference in these percentages (*p* > 0.84). By contrast, Map B characterized responses 12.1 ± 2.7% of the time, which was significantly less than either Map A (t_(115)_ = 5.71; p < 0.001) or Map C (t_(115)_ = 5.51; *p* < 0.001). Given this pattern of results, we considered the percentage of time responses were characterized by Map A as representative of typical infantile processing; “TIP”.

We next assessed how TIP was associated with behavioural outcomes. First, the degree of prematurity at birth (indexed by GA) was a reliable predictor of the how often Map A characterized stimulus processing over the 176–231 ms post-stimulus time window (*p* < 0.001 for all multivariable regression analyses). For example, the probability of an infant born at 25 weeks GA of having ERP responses predominantly characterized by Map A is 27%, whereas it rises in a linear fashion to 60% for an infant born at 40 weeks GA. Noting that Map A not only characterized responses to multisensory stimuli, but also to the summed unisensory condition, we examined this correlation when controlling for the percentage of time Map A characterized an infant’s response to the summed unisensory condition. It was no longer associated (partial *r* = 0.09, *p* = 0.5).

We next assessed the extent to which stimulus processing near birth, as indexed by the relative preponderance of Map A in characterizing ERP responses to the multisensory stimulus over the 176–231 ms post-stimulus time window, was predictive of the typicality of sensory reactivity in the home measured at 12 months of age with the ITSP. This was indeed the case regardless of whether GA or postnatal days (PND) of test was statistically controlled (Table 2). To further assess the specificity of the association, we examined the same association after statistically controlling the relative preponderance of Map A in characterizing ERP responses to the summed unisensory condition. The partial correlation was significant (partial r = − 0.3, *p* = 0.04).

**Table 2:** Associations between EEG multisensory measures and behavioral outcomes

	Unadjusted OR^a^ [CI]	Adjusted for GA OR^a^ [CI]	Adjusted for PND OR ^a^ [CI]
Odds ratio of typicality of sensory reactivity at 12 months of age	0.26 [0.07–0.96]*	0.23 [0.06–0.86]*	0.21 [0.06–0.80]*
Odds ratio for internalizing tendencies above threshold at 24 months of age	0.22 [0.06–0.83]*	0.24 [0.06–0.91]*	0.25 [0.07–0.93]*
Odds ratio for externalizing tendencies above threshold at 24 months of age	0.50 [0.1–2.54]	0.51 [0.09–2.72]	0.49 [0.09–2.57]

The top row shows unadjusted and adjusted odds ratio of typicality of sensory reactivity at 12 months of age as a function of the preponderance of Map A characterizing brain activity in response to multisensory stimuli. The lower two rows show unadjusted and adjusted odds ratio of internalizing and externalizing group membership at two years as a function of the preponderance of Map A characterizing brain activity in response to multisensory stimuli

*GA* gestational age at birth, *PND* postnatal days

^a^Odds ratios (OR) less than 1 indicate that children have a greater probability of having typical sensory reactivity, typical internalizing tendencies, and typical externalizing tendencies

*p < 0.05

Finally, we assessed whether stimulus processing near birth, again indexed by the relative preponderance of Map A in characterizing ERP responses over the 176–231 ms post-stimulus time window during the multisensory condition, was predictive of internalizing and externalizing group membership at 24 months of age as measured by the CBCL. Infants whose brain activity was more often characterized by Map A had lower odds of having internalizing scores above risk thresholds, regardless of whether GA or PND was statistically controlled. To examine whether this association was specific to the multisensory condition, we tested the association after controlling for the relative preponderance of Map A in characterizing ERP responses over the 176–231 ms post-stimulus time window during the summed unisensory condition. This association was significant, partial *r* = − 0.39, *p* = 0.006. There was no similar evidence in the case of externalizing tendencies (Table 2).

## Discussion

We provide the first electrophysiologic characterization of auditory-somatosensory multisensory processing in full-term and preterm neonates at their discharge from the hospital. Multisensory processing in full-term neonates was characterized by a linear addition of unisensory signals and by the preponderance of a single ERP topography for responses both to multisensory stimulus pairs and summed responses to unisensory conditions—what we refer to as “typical infantile processing” or TIP. By contrast, multisensory processing in preterm infants was characterized by non-linear neural response interactions and less frequent TIP, with responses more often characterized by additional ERP topographies. We further showed that multisensory processes, as indexed by TIP, were a reliable predictor of typical sensory thresholds when the child, independently of preterm status, was aged 12 months, as well as the absence of internalizing behavioral tendencies at 24 months. This was the case even after controlling for contributions of GA, postnatal age, and unisensory processes. This overall pattern of results highlights the importance of the establishment of the neural networks that support typical multisensory processes during early life and how the integrity of these networks and processes may foretell later sensory and clinical measures.

Together, these data provide evidence supporting the construct validity of topographic ERP analyses as a measure of the typicality of multisensory processing in neonates. Additionally, they support the hypothesis that one reason children with preterm birth tend to experience more internalizing symptoms may be at least partially grounded in the fact that they process multisensory stimuli differently as compared with their full-term peers. To understand the implications of our findings, it is useful to place them into the greater context of what is known about multisensory integration in older children, as no other study has examined brain-based measures in response to multisensory stimuli this early in development.

### Multisensory Processes Across the Lifespan

The first set of results in our study centers on multisensory interactions in response to the combination of audition and touch. While full-term infants exhibited a linear response to such combinations, such that there were no differences between the responses to multisensory and summed unisensory conditions, preterm infants exhibited non-linear neural response interactions that began at approximately 145 ms post-stimulus onset. Moreover, this non-linearity was the result of topographic differences between responses to multisensory and summed unisensory conditions (see Figs. 2 and 3). These findings need to be considered against the backdrop of findings in older infants, aged 6–12 months (Stephen et al. 2007), older children, aged 6–13 years (Brett-Green et al. 2008; Russo et al. 2010), as well as adults (Foxe et al. 2000; Murray et al. 2005). In a small pilot study of 9 infants, (Stephen et al. 2007) claimed that non-linear response interactions were larger in the subset of four 11–13 month-olds than in five 6–9 month-olds, suggesting a developmental increase in these non-linear effects. Although there was no explicit reporting of the timing or topography of these effects, visual inspection of their data suggests that these effects are present during the initial 100–150 ms post-stimulus onset in both age groups (cf. their Figs. 1 and 2). These data appear consistent with data that has been obtained from older children, who exhibit non-linear neural response interactions within the first 100–150 ms post-stimulus onset (Brett-Green et al. 2008). Such effects appear accelerated in adults, in which these interactions are seen within the first 50–90 ms post-stimulus onset (Murray et al. 2005). Collectively, these results suggest that non-linear auditory-somatosensory neural response interactions emerge within the first year of life. The present results build on this literature by showing that non-linear neural response interactions are not observable in full-term neonates at the time of their discharge from the hospital. Robust ERPs were observable in response to stimuli from each sensory modality alone, and the response to multisensory stimulus pairs was equivalent to the summed responses to each constituent stimulus alone. In these full-term neonates, there was no evidence for non-linear neural response interactions nor evidence for topographic differences between responses to multisensory pairs and summed unisensory conditions. One strong implication of these results is that early-life experience is likely to be a major contributor to the emergence of non-linear neural response interactions as neonates transition into infancy.

Our observation of robust non-linear neural response interactions in our group of preterm neonates who were recorded at an equivalent GA as the full-term neonates, lends support to the idea of early-life experience as a major contributor to the development of multisensory processes. As detailed above, the sensory experiences of preterm neonates dramatically differ from those of full-term neonates. Studies in developing animals have shown how strongly early-life experiences can shape patterns of multisensory integration. For example, animals reared under conditions where multisensory events were temporally synchronous but spatially separated results in robust multisensory integration to these pairings – a finding strikingly different from circumstances in which animals are reared with temporally and spatially contiguous stimuli events (Wallace and Stein 2007).

In addition to the contributions of atypical early-life experiences, it must also be acknowledged that the preterm brain is more immature at birth when compared with the full-term brain. We therefore cannot readily disentangle whether preterm infants, at the time of discharge from hospital, are prone to manifest multisensory processing because they are now on a distinct developmental trajectory and/or because they are exhibiting accelerated maturation of an otherwise intact process. However, two aspects of the results are worth noting. First, the multisensory processes we observed in the preterm group were the result of topographic differences between responses to multisensory and summed unisensory conditions, which differs from the findings in adults in which multisensory interactions are driven almost exclusively by differences in response strength (Murray et al. 2005; Sperdin 2009). That is, auditory-somatosensory multisensory interactions in adults are characterized by the responses of a common brain network, whereas in preterm infants these processes appear dependent upon the activity within multiple networks. Comparable topographic analyses have not been conducted to date or reported in children. Second, the extent to which the topography of a preterm neonate’s brain responses were characterized by the standard topography, what we refer to as TIP, directly related to the degree of prematurity. This would suggest that brain maturation itself contributes to the patterns of multisensory processes we have observed. Given the immaturity of these preterm infants’ brains and coupled with their altered sensory environments, we propose that the development of the multisensory processes observed here are likely indicative of a distinct trajectory rather than simply an acceleration of what is otherwise neurotypical. However, we would hasten to add that additional data, including in utero functional data, would be required to support or refute this proposition with any level of conviction. However, there is now mounting quantitative investigation of the altered sensory experience in the NICU versus home environment (Liszka et al. 2020) as well as of the potential benefical effects of sensory enrichment (Webb et al. 2015). Nonetheless, our proposition that the multisensory responses we observed are the result of immature brain circuits and atypical sensory experience receives some measure of support in the set of correlational analyses that establish links between brain function and later outcome measures.

### Associations Between Multisensory Processes and Later Developmental Outcomes

The second set of results concerns how multisensory brain responses, as characterized by their topography and more specifically by TIP, are predictive of later sensory thresholds and internalizing tendencies. These results were obtained across the cohort of both full-term and preterm infants. Thus, this brain-based functional index of multisensory processing appears to be particularly informative about the later integrity of other neurodevelopmental outcomes. This is in agreement with a growing body of evidence showing that multisensory processes may be particularly informative about the integrity of higher-level functions, and likely scaffold the development of these higher-order abilities. For example, Denervaud et al. (2020) showed in schoolchildren that multisensory gains on a simple detection task are predictive of both working memory and fluid intelligence measures, even when controlling for age. Likewise, Murray et al. (2018) used a similar multisensory detection task to accurately classify healthy ageing from older individuals with mild cognitive impairment. In these studies at opposite ends of the lifespan, the effects were specific for multisensory processes; unisensory processes were ineffective predictors of these cognitive functions. In the current work we extend these links between functional multisensory measures to later developmental indices that measure sensory thresholds at one age (12 months) and internalizing tendencies at a later age (24 months).

It must be pointed out here that correlational analyses cannot exclude the possibility that additional variables might also explain the associations of interest. Consequently, we cannot draw inference regarding causality here, even if implied by the longitudinal design. Nonetheless, our study’s findings are consistent with a hypothesis that atypical multisensory processing may contribute to greater internalizing symptoms 24 months of age; a plausible view given that the multisensory stimulation received in early life (in an environment such as a NICU) may be a negative experience for some newborns. Because our cohort includes a large proportion of preterm infants, most atypical behavioral tendencies at 2 years of age are on the internalizing or dysregulated end of the spectrum instead of the externalizing one, consistent with published literature (Spittle et al. 2009). Internalizing tendencies are often indicative of withdrawal or fearful behaviors, with both passive and active hypersensitivity reactions. These concepts overlap with sensory processing frameworks that describe children’s neurological threshold to environmental stimuli in their home or school environment: in these models, infants with low neurological thresholds to sensory stimuli can adapt to their threshold using active strategies such as avoiding (withdrawing from them) or passive sensitivity. However, the hypothesis that construction of multisensory responses in infancy contributes to atypical sensory reactivity and later internalizing tendencies can be better assessed using an experimental research design that manipulates multisensory processing and follows the infants for 24 months to assess internalizing symptoms.

### Limitations

This study’s main limitations include its correlational nature and size. Although the largest study of its kind, it was not designed to investigate causality or maturation, which would require longitudinal follow-up using consistent EEG paradigms throughout. Additionally, follow-up of the cohort to 2 years of age proved challenging in a large rural area (> 100,000 km^2^) with high population mobility and highly variable health-insurance status influencing access to clinically-indicated follow-up services. Approximately 10% of Tennesseans had no health insurance in 2018, and about half the population receives job-based coverage through an employer instead of federally-provided insurance (Pellegrin 2020). Therefore, future confirmatory research is needed to determine the replicability of these findings.

## Conclusion

We showed that the integrity of sensory and multisensory processes in full-term and preterm neonates can be quantitatively studied using high-density EEG and topographic analyses, showcasing the versatility of EEG techniques (Biasiucci et al. 2019). Future studies could use the proposed measure of typicality of multisensory processes (TIP) to determine whether interventions targeting multisensory processes affect functional outcomes, through improved typicality of the brain’s response to (multisensory) stimuli. Still, this remains the first study to offer evidence for the very early emergence of multisensory processes in human infants, whether born full-term or preterm, and their association with later sensory reactivity and behavioral problems throughout the lifespan.

## Electronic supplementary material

Below is the link to the electronic supplementary material.

Supplementary file1 (PDF 453 kb)

## Data Availability

All reasonable requests from qualified scientists for unique research resources (ERP paradigms, protocols and expertise) developed with NIH funds for research purposes will be honored. We will fill requests in a timely manner. We will adhere to the NIH Grant Policy on Sharing of Unique Research Resources including the Sharing of Biomedical Research Resources Principle and Guidelines for Recipients of NIH Grants and Contracts. There is no unique biological information that could be made available to the scientific community. De-identified ERP raw data will be retained on the Abigail Wexner Research Institute at Nationwide Children’s Hospital server, and assessment data will be retained in REDCap. These data will be made available to investigators who make specific inquiry for good cause 5 years after the conclusion of the final outcomes.
