# Supplementary material for: Neonatal Multisensory Processing in Preterm and Term Infants Predicts Sensory Reactivity and Internalizing Tendencies in Early Childhood
Source: Brain Topogr. 2020 Aug 12;33(5):586–99. doi: 10.1007/s10548-020-00791-4 (PMC7429553; doi:10.1007/s10548-020-00791-4)
Supplement: Supplementary file 1 — Supplementary file1 (PDF 453 kb) [file 10548_2020_791_MOESM1_ESM.pdf]

Supplementary Appendix

Neonatal Multisensory Processing in Preterm and Term Infants Predicts Sensory Reactivity and Internalizing Tendencies in Early Childhood

Nathalie L. Maitre, Alexandra P. Key, James C. Slaughter, Paul J. Yoder, Mary Lauren Neel, Céline Richard, Mark T. Wallace, Micah M. Murray

Supplementary Material

For the later time window (232-375 ms), Maps D-F were fitted to the data. The ANOVA revealed a significant main effect of Map ($F_{(1.73,197.60)}$=15.38; p<0.001; η_p^2=0.12), a non-significant trend for a Condition × Map interaction ($F_{(1.88,214.16)}$=2.43; p=0.094; η_p^2=0.02), as well as a significant Preterm Status × Map interaction ($F_{(1.73,197.60)}$=12.93; p<0.001; η_p^2=0.10). The 3-way interaction was not significant (p>0.10). In the same manner as with the results during the earlier time window, we probed the non-significant trend with separate 1-way ANOVAs for each condition, collapsing across Preterm Status. For the multisensory pair condition, there was an effect of Map ($F_{(1.85,212.68)}$=9.51; p<0.001; η_p^2=0.08). Map D predominantly characterized responses to multisensory stimulus pairs. On average, Map D best correlated with individual infant's brain responses 49.3±4.1% of the time (mean±s.e.m.), which was significantly more than either Map E (23.3±3.2%; $t_{(115)}$=4.06; p<0.001) or Map F (27.4±3.7%; $t_{(115)}$=3.06; p<0.003); the latter two of which did not differ (p>0.45). For the summed unisensory condition, there was an effect of Map ($F_{(1.72,197.66)}$=9.26; p<0.001; η_p^2 =0.08). Map D and Map F characterized responses 44.2±4.0% and 37.6±4.0% of the time, respectively, with no difference in these percentages (p>0.38). By contrast, Map E characterized responses 18.2±2.8% of the time, which was significantly less than either Map D ($t_{(115)}$=4.57; p<0.001) or Map F ($t_{(115)}$=3.51; p<0.001).

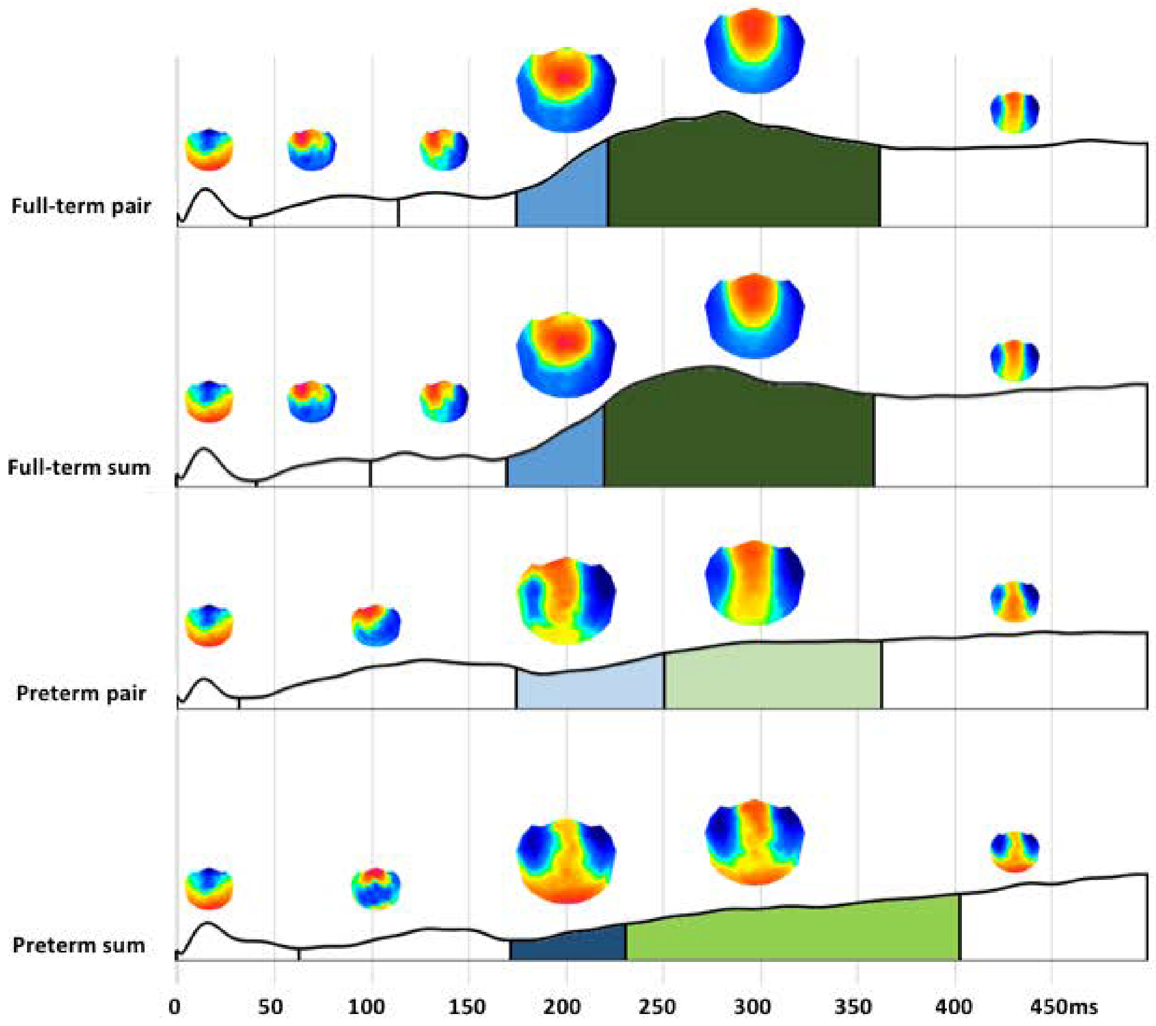

Supplementary Fig. 1. Results of the topographic clustering. The waveforms indicate the GFP of the group-averaged data for a given group and condition. The topographic maps are displayed flattened with nose upwards and the left hemiscalp on the left. Colored time periods refer to template maps Map A, Map B, and Map C (blue hues; see Figure 2) and Map D, Map E, and Map F (green hues; see Supplementary Figure 2).

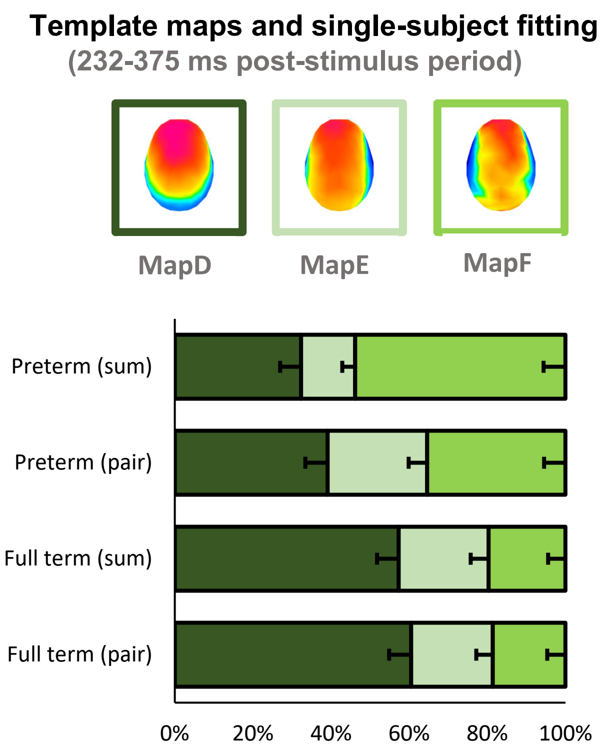

Supplementary Fig. 2. Template maps identified over the 232-375 ms post-stimulus period via unsupervised hierarchical clustering of the ERP topography using the group-averaged dataset concatenated across conditions and full term and preterm infants. The maps are displayed with the nose upwards and left hemiscalp on the left. The bar graphs display the single-subject fitting based on the spatial correlation between the template maps shown in a and each time point of the infant's data over the 232-375 ms post-stimulus period. The bars show the average percentage of time each template map yielded the highest spatial correlation (error bars indicate s.e.m.).
